# Association between early childhood caries and anthropometric growth and nutritional status in preschool children: a cross-sectional study

**DOI:** 10.3389/fped.2025.1623666

**Published:** 2025-11-19

**Authors:** Zhenzhen Cao, Ran Yu, Jingru Ma, Ying Zhang, Xi Dong, Lixin Fan

**Affiliations:** 1 Department of Child Health Care, Shijiazhuang Maternal and Child Health Hospital, Shijiazhuang, Hebei Province, China; 2 Reproductive Medicine Department, Shijiazhuang Maternal and Child Health Hospital, Shijiazhuang, Hebei Province, China; 3 Outpatient Department of Shijiazhuang Maternal and Child Health Hospital, Shijiazhuang, Hebei Province, China

**Keywords:** preschool children, dental caries, growth and nutritional status, early childhood caries (ECC), developmental delay

## Abstract

**Objective:**

To estimate the prevalence and dmft burden of early childhood caries (ECC) among preschool children and to examine associations between ECC and anthropometric growth indicators (HAZ, WAZ, WHZ) as well as behavioral and parental factors.

**Methods:**

We conducted a cross-sectional study of 380 children aged 3–6 years selected via multistage cluster sampling from 15 kindergartens in Shijiazhuang, China. ECC was assessed according to WHO Oral Health Survey methods, and dmft (decayed, missing, and filled primary teeth) was calculated. Anthropometric growth was evaluated using WHO *Z*-scores: height-for-age (HAZ), weight-for-age (WAZ), and weight-for-height (WHZ), with standard cutoffs (*Z* < −2 indicating stunting, underweight, and wasting, respectively). Parents completed structured questionnaires on feeding patterns (including night feeding), frequency of sweet-food consumption, bedtime toothbrushing, use of fluoride toothpaste, annual oral examinations, vitamin D and calcium supplementation, and parental oral-health knowledge. Group comparisons used chi-square and *t*-tests; multivariable logistic regression identified factors associated with ECC; Spearman correlation assessed associations between caries count and growth *Z*-scores.

**Results:**

ECC prevalence was 58.68% (223/380), and mean dmft was 2.34 ± 2.35, with no significant differences by sex or age group. In adjusted models, higher sweet-food frequency was associated with greater odds of ECC (OR 1.72, 95% CI 1.19–2.49). Protective factors included vitamin D supplementation for ≥2 years (OR 0.40, 95% CI 0.18–0.86), bedtime toothbrushing (OR 0.22, 95% CI 0.13–0.38), use of fluoride toothpaste (OR 0.04, 95% CI 0.01–0.13), annual oral examinations (OR 0.26, 95% CI 0.15–0.44), and qualified parental oral-health knowledge (OR 0.38, 95% CI 0.23–0.65). Children with ECC had higher proportions of stunting, underweight, and wasting than those without ECC. Caries count was inversely correlated with WHZ (*r* = −0.649, *P* < 0.001).

**Conclusion:**

In this population, ECC was common. Modifiable behaviors and parental knowledge were strongly associated with ECC, and greater caries severity was linked to lower WHZ, suggesting a relationship between ECC and acute nutritional status. Strengthening sugar intake control, bedtime toothbrushing, fluoride use, and routine dental examinations may help prevent ECC and mitigate its potential impact on growth.

## 1 Introduction

Dental caries is a biofilm-mediated, sugar-driven, multifactorial disease that leads to demineralization and destruction of tooth hard tissues (1). When it occurs in the primary dentition of preschool children, it is termed early childhood caries (ECC) (2). The morphology and mineralization of primary teeth, coupled with behavioral and dietary patterns in early life, render preschool children particularly susceptible to ECC (3, 4). Globally, a substantial proportion of children are affected, with a pooled prevalence of approximately 57.3% among those aged 3–6 years (5, 6). In China, reported prevalence among preschoolers ranges from 59.0% to 74.3%, and the mean dmft burden has shown an upward trend in some regions (3, 7, 8).

Beyond its high prevalence, ECC adversely affects children's oral and general health. Untreated lesions can lead to pain, pulp infection, and abscess formation; impair mastication and sleep; and reduce dietary intake, thereby diminishing oral health–related quality of life (9–12). Premature loss of primary teeth may disrupt occlusion and oral function and has been associated with suboptimal nutritional status (13, 14). These pathways provide biological plausibility for a link between ECC and growth.

However, evidence regarding the relationship between ECC and children's growth is inconsistent. Studies have reported positive, negative, or null associations between ECC and anthropometric outcomes, likely reflecting differences in study design, populations, and control of confounding factors (15–19). Data from mainland China that concurrently characterize ECC, anthropometric growth indicators, and behavioral determinants in preschool children remain limited.

Therefore, this cross-sectional study aimed to: (i) estimate the prevalence of ECC and the dmft burden among preschool children in Shijiazhuang, China; (ii) compare anthropometric growth indicators—height-for-age (HAZ), weight-for-age (WAZ), and weight-for-height (WHZ)—between children with and without ECC; (iii) examine the association between caries count and these WHO Z-scores; and (iv) identify behavioral and parental factors associated with ECC using multivariable analysis.

## 2 Materials and methods

### 2.1 Study subjects

In this study, a multi—stage cluster sampling method was employed. Fifteen kindergartens in Shijiazhuang, Hebei Province were selected. These kindergartens were numbered and chosen using a simple randomization method based on a computer—generated random sequence. Ultimately, 380 preschool children from the 15 selected kindergartens were included in this study.

Inclusion criteria:

1. Aged between 3 and 6 years old;
2. Directly raised by their parents;
3. For children classified into the ECC group, dental caries in the primary dentition was diagnosed according to the WHO Oral Health Surveys: Basic Methods (5th edition). A tooth was considered carious when a cavitated lesion at the dentine level was detected on visual–tactile examination. Non-cavitated enamel demineralization/white spot lesions were not counted as caries for the purpose of dmft calculation or ECC case definition. ECC was defined as having dmft ≥ 1 in children under 6 years of age (5);
4. Availability of complete anthropometric examination data, defined as valid measurements of age in months, sex, body weight, and body height obtained on the same visit, following standardized procedures described below, with no missing values and passing internal quality checks.

Exclusion criteria:

1. Those who had used antibiotics within 1 month before the examination or had used hormones and immunosuppressants within 6 months;
2. Those suffering from immune system diseases that promote the decay of primary teeth;
3. Those with a history of dental diseases such as enamel hypoplasia and pulpitis;
4. Children with diseases in the digestive, endocrine systems or metabolic—related diseases;
5. Those from families with low height, low weight, or obesity.

This study adhered to the principles of the Declaration of Helsinki and was approved by the Ethics Review Committee of Shijiazhuang Maternal and Child Health Hospital. All guardians of the children were informed about the content of this study, voluntarily participated, and signed the informed consent forms related to this study.

### 2.2 Sample size calculation

Based on the preliminary results of previous studies, the prevalence of ECC among Chinese preschool children is approximately 74% (20). According to the sample—size calculation formula, to estimate a 10% width of the 95% confidence interval, approximately 295 children are required [*n* = 4 × 1.96^2^ × *P* × (1 − *P*)/*L*^2^, where *n* is the number of participants, *P* is the estimated disease prevalence, and *L* is the width of the confidence interval, which is 0.10 in this study]. Assuming an 80% response rate, at least 369 children should be initially recruited. In this study, 380 preschool children were finally recruited as the study subjects.

### 2.3 Questionnaire and covariates

Parents or primary caregivers completed a structured questionnaire covering:

Child basic information: age (months), sex, only child (yes/no), recent morbidity (past 2 weeks).

Feeding history: exclusive breastfeeding (first 6 months; yes/no), duration of breastfeeding (months), bottle feeding (yes/no), night-time feeding during infancy (frequency per night), night-time feeding after tooth eruption (never/occasionally/≥3 nights per week).

Current diet (past 3 months) using a brief food frequency module: frequency of sweet foods (candies, cookies, cakes, chocolates) and sugar-sweetened beverages (sodas/juices/sweetened milk/tea) categorized as never/≤1 time per week/2–4 times per week/≥5 times per week; frequency of fruit and vegetable intake (days per week), dairy intake (servings per day), and protein-rich foods (meat/eggs/beans; days per week).

Oral hygiene: toothbrushing frequency (per day), bedtime toothbrushing (yes/no), use of fluoride toothpaste (yes/no), initiation age of toothbrushing (years), dental visits in the past 12 months (yes/no).

Micronutrient supplementation: vitamin D and calcium (ever use: yes/no), current use (yes/no), cumulative duration (<6 months/6–<12 months/12–<24 months/≥24 months), typical daily dose if known, and source (drops/tablets/formula).

Parental information: parental age, education level, and self-rated oral health knowledge; parental oral-health knowledge score derived from 10 items (correct = 1, incorrect/unknown = 0), summed to a total score (0–10); a score ≥7 was considered “qualified” knowledge.

The full questionnaire (English translation) has been provided as Supplementary Material (Supplementary File S1), including item wording, response options, and coding rules.

### 2.4 Oral examination

Two trained dentists performed visual–tactile examinations in the kindergarten setting following the WHO Oral Health Surveys: Basic Methods (5th edition). Examinations were conducted under adequate lighting using disposable plane mouth mirrors and WHO CPI probes; cotton rolls/gauze were used to remove debris and gently dry tooth surfaces when needed. Caries experience was recorded at the tooth level using the dmft index, where dmft = d + m + f: d = number of carious teeth with cavitated lesions into dentine (including teeth with restorations accompanied by secondary caries), m = number of teeth missing due to caries (excluding teeth lost due to trauma or orthodontic reasons), and f = number of filled teeth without secondary caries. We did not classify lesions as “active” or “arrested” beyond the WHO diagnostic threshold. White spot lesions (initial, non-cavitated) were not included in dmft and were not used to define ECC status (21).

### 2.5 Growth and development assessment

Age (months), sex, standing height (cm), and body weight (kg) were collected during the same visit. Height was measured to the nearest 0.1 cm using a wall-mounted stadiometer with children barefoot and standing upright; weight was measured to the nearest 0.1 kg using a calibrated digital scale with light clothing. Two measurements were taken for each parameter; if they differed by >0.5 cm (height) or >0.2 kg (weight), a third measurement was obtained, and the two closest values were averaged. Anthropometric Z-scores were computed using WHO growth standards: Among them, HAZ < −2 indicates short stature, −2 ≤ HAZ ≤ 2 indicates normal stature, and HAZ > 2 indicates tall stature; WAZ < −2 indicates low weight, −2 ≤ WAZ ≤ 2 indicates normal weight, and WAZ > 2 indicates overweight; WHZ < −2 indicates underweight, −2 ≤ WHZ ≤ 2 indicates normal weight, and WHZ−>−2 indicates obesity (22).

### 2.6 Outcomes and prevalence calculation

ECC was defined as dmft ≥ 1. ECC prevalence was calculated as the number of children with ECC divided by the total number examined.

### 2.7 Statistical analysis

In this study, SPSS 25.0 software was used for statistical analysis. Measurement data were expressed as mean ± standard deviation. The independent—samples *t*-test was used for comparisons between two groups, and one—way analysis of variance was used for comparisons among multiple groups. Count data were expressed as the number of cases and percentages, and the X^2^-test was used for comparisons between groups. Multivariate logistic regression analysis was used for the analysis of influencing factors, and Spearman analysis was used for correlation analysis. A *P*-value < 0.05 was considered statistically significant. Details of variable coding (e.g., sweet-food frequency categories) are provided in Supplementary File S1.

## 3 Results

### 3.1 Analysis of ECC in preschool children

**Figure 1:**
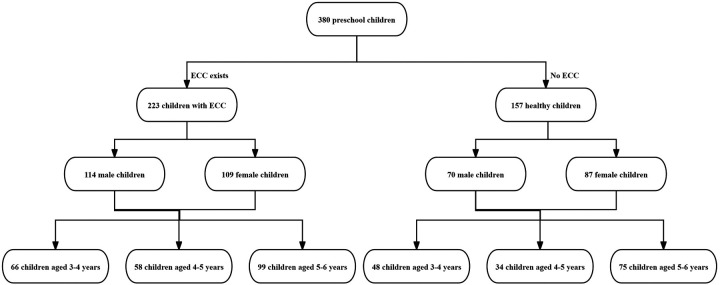
Status of early childhood caries (ECC) in preschool children.

In this research, a total of 380 preschool children were enlisted. The legal guardians of all the recruited children gave their written informed consents, and all the children went through oral inspections. Among them, 157 children who did not have early childhood caries (ECC) were placed in the healthy group, whereas 223 children with ECC were incorporated into the ECC group. The prevalence rate of ECC was 58.68%. Among the children being examined, the aggregate number of decayed teeth was 889, and the average number of decayed teeth was (2.34 ± 2.35). No statistically notable disparities were found in the prevalence of ECC and the average number of decayed teeth between different genders (*P* > 0.05). Moreover, no statistically significant differences were detected in the prevalence of ECC and the average number of decayed teeth across various age groups (*P* > 0.05) (Table 1, Figure 1).

**Table 1:** Analysis of early childhood caries (ECC) in preschool children.

Group	Number of cases	Number of children with ECC	Mean DMFT
Number of cases in males	184	114 (61.96%)	2.41 ± 2.38
Number of cases in females	196	109 (55.61%)	2.30 ± 2.33
*t*/X^2^		1.576	0.449
*P*		0.210	0.654
Number of cases aged 3–4 years	114	66 (57.89%)	2.46 ± 2.52
Number of cases aged 4–5 years	92	58 (63.04%)	2.31 ± 2.22
Number of cases aged 5–6 years	174	99 (56.90%)	2.29 ± 2.36
*F*		0.980	0.2990
*P*		0.613	0.742

### 3.2 Comparative analysis of general data between children with ECC and normal children

**Figure 2:**
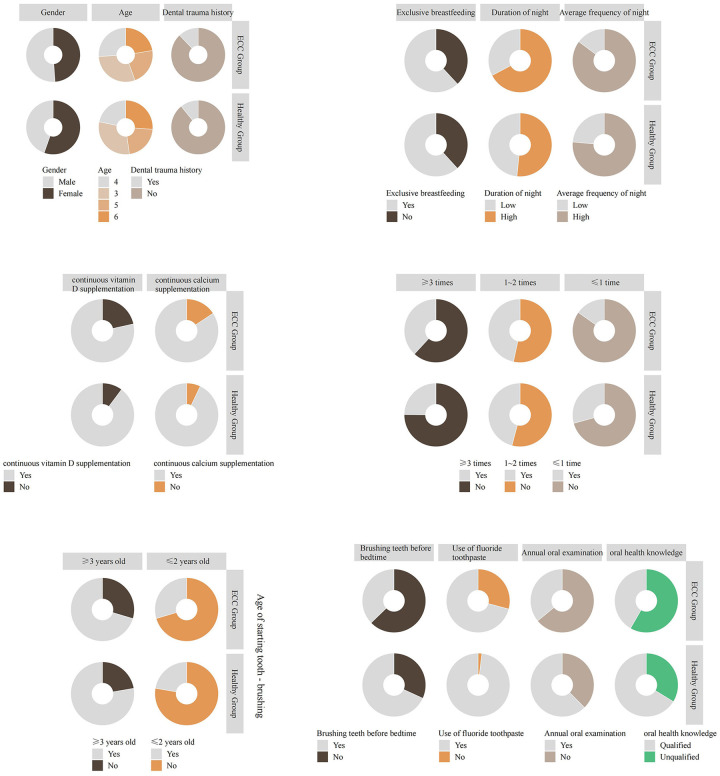
Comparative analysis of general information between children with ECC and normal children.

In univariate comparisons, children with ECC differed significantly from caries-free peers across feeding, hygiene, and parental factors. The ECC group reported a longer duration of night-time feeding and a higher mean number of night feeds (both *P* < 0.001), as well as a higher daily frequency of sweet-food consumption over the past year (*P* = 0.0011). By contrast, the proportions with continuous vitamin D supplementation for ≥2 years (*P* = 0.0037) and continuous calcium supplementation for ≥2 years (*P* = 0.0106) were lower in the ECC group. Bedtime toothbrushing, use of fluoride toothpaste, and receipt of an annual oral examination were also less common among children with ECC (all *P* < 0.001). In addition, parents of caries-free children were more likely to achieve a qualified level of oral-health knowledge (*P* < 0.001) (Table 2, Figure 2).

**Table 2:** Characteristics of children with and without ECC (univariate comparisons).

Indicator		ECC group (*n* = 223)	Healthy group (*n* = 157)	*t*/X^2^	*P*
Gender	Male	114	70	1.5755	0.2094
	Female	109	87		
Age		4.37 ± 1.13	4.43 ± 1.18	0.5079	0.6118
Dental trauma history		27	17	0.1473	0.7011
Exclusive breastfeeding within 1 year of age	Yes	138	97	0.0004	0.9840
	No	85	60		
Duration of night—feeding (months)		12.42 ± 4.82	10.58 ± 3.27	4.1590	<0.001
Average frequency of night—feeding		2.78 ± 1.19	2.31 ± 1.03	3.9648	<0.001
Currently, continuous vitamin D supplementation for ≥2 years		175	141	8.4500	0.0037
Currently, continuous calcium supplementation for ≥2 years		188	146	6.5372	0.0106
Average daily frequency of sweet food intake in the past year	≥3 times	85	39	13.6307	0.0011
	1∼2 times	104	72		
	≤1 time	34	46		
Brushing teeth before bedtime		83	107	35.2638	<0.001
Use of fluoride toothpaste		158	154	46.5209	<0.001
Age of starting tooth—brushing	≥3 years old	157	122	2.5183	0.1125
	≤2 years old	66	35		
Annual oral examination	Yes	80	98	26.8203	<0.001
	No	145	59		
Parents' cognitive level of oral health knowledge	Qualified	93	104	22.2203	<0.001
	Unqualified	130	53		

Data are *n* (%) unless otherwise indicated. *P*-values from chi-square tests (categorical) or *t-*tests/Mann–Whitney *U-*tests (continuous). Variable definitions and coding detailed in Supplementary File S1. ECC defined as dmft ≥1 (WHO criteria; white spot lesions excluded).

### 3.3 Analysis of influencing factors for the onset of ECC

A multivariate Logistic regression analysis was conducted with the presence or absence of ECC as the dependent variable and the variables with significant differences in Table 2 as independent variables. The results showed that the average daily frequency of consuming sweet foods was an independent risk factor for the occurrence of dental caries. In contrast, continuous vitamin D supplementation for ≥2 years, brushing teeth before bedtime, using fluoride toothpaste, having annual oral examinations, and having a qualified cognitive level of oral health knowledge were protective factors for preventing dental caries (Table 3).

**Table 3:** Analysis of influencing factors for the onset of ECC.

Independent variable	*β*	S.E.	Wald	*P*	95% CI
Duration of night—feeding	0.060	0.044	1.820	0.177	0.973–1.159
Frequency of night-time milk feeding	0.221	0.167	1.752	0.186	0.899–1.732
Continuous vitamin D supplementation for 2 years or more	−0.927	0.398	5.440	0.020	0.181–0.862
Continuous calcium supplementation for 2 years or more	−0.622	0.464	1.794	0.180	0.216–1.334
Average daily intake of sweet foods	0.543	0.188	8.359	0.004	1.191–2.485
Brushing teeth before bedtime	−1.512	0.273	30.705	<0.001	0.129–0.376
Use of fluoride toothpaste	−3.291	0.647	25.883	<0.001	0.010–0.132
Annual oral examination	−1.353	0.275	24.209	<0.001	0.151–0.443
Satisfactory level of knowledge about oral health	−0.960	0.270	12.678	<0.001	0.226–0.649

### 3.4 Analysis of the growth and development status of preschool children

**Figure 3:**
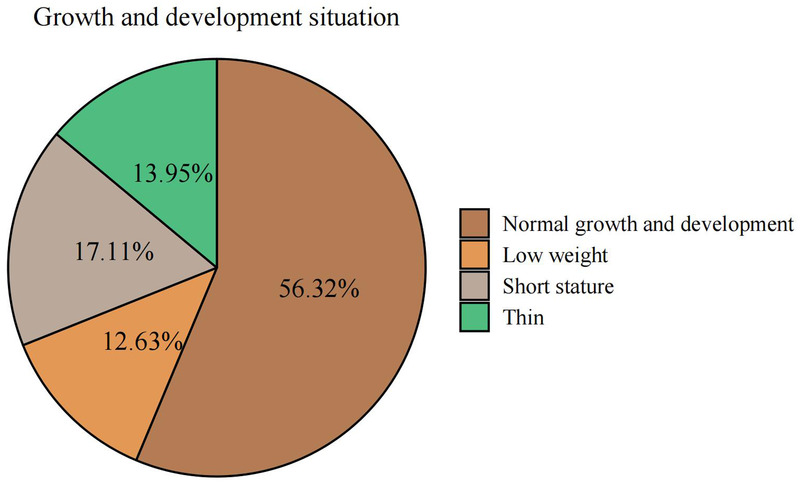
Analysis of growth and development status of preschool children.

Among the 380 examined preschool children, 214 (56.32%) had normal growth and development, 48 (12.63%) had low body weight, 65 (17.10%) had short stature, and 53 (13.95%) were underweight (Table 4, Figure 3).

**Table 4:** Analysis of growth and development Status of preschool children.

Growth and development situation	Number of cases
Normal growth and development	214 (56.32%)
Low weight	48 (12.63%)
Short stature	65 (17.10%)
Thin	53 (13.95%)

### 3.5 Comparative analysis of growth and development status between children with ECC and normal children

**Figure 4:**
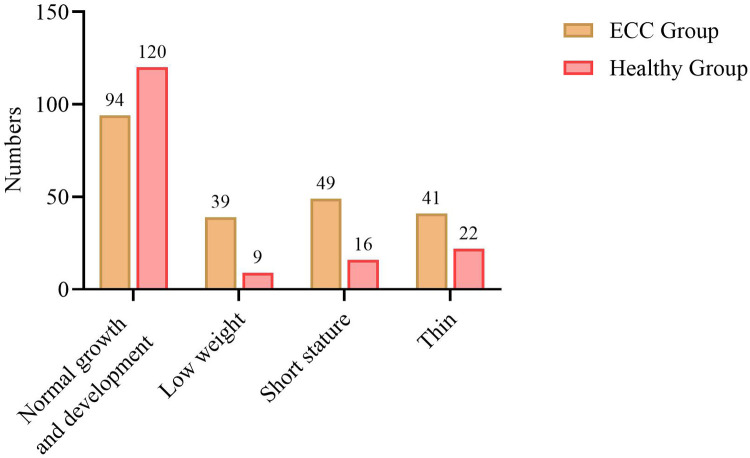
Growth and development status of children with ECC.

Among the 380 examined children, 49 children with dental caries had short stature, accounting for 75.38% of all short—statured children; 39 children with dental caries had low body weight, accounting for 81.25% of all low—weight children; and 41 children with dental caries were underweight, accounting for 77.36% of all underweight children. The proportions of short—statured, low—weight, and underweight children in the dental caries group were higher than those in the healthy group (Table 5, Figure 4).

**Table 5:** Growth and development Status of children with ECC.

Growth and development situation	ECC group (*n* = 223)	Healthy group (*n* = 157)	X^2^	*P*
Normal growth and development	94 (42.15%)	120 (56.07%)	44.011	<0.001
Low weight	39 (81.25%)	9 (18.75%)	11.539	<0.001
Short stature	49 (75.38%)	16 (24.62%)	9.020	0.003
Thin	41 (77.36)	12 (22.64)	8.859	0.003

### 3.6 A nalysis of the relationship between the severity of ECC and the growth and development status of children with dental caries

The results of Spearman's correlation analysis showed that there was a negative correlation between the number of dental caries and the Weight—for—Height Z-score (WHZ) in preschool children (*r* < 0, *P* < 0.05) (Table 6).

**Table 6:** Relationship between the severity of ECC and the growth and development Status of children with dental caries.

ECC	WAZ	WHZ	HAZ
*r*	−0.010	−0.649	0.069
*P*	0.886	<0.001	0.317

## 4 Discussion

This study examined the association between early childhood caries (ECC) and growth status among preschool children, and identified modifiable behavioral and parental factors linked to ECC. ECC remains under-recognized and undertreated in many settings (23). Three main findings emerged. First, the prevalence of ECC and the mean dmft in our sample were high. Second, higher frequency of sweet-food intake was associated with greater odds of ECC, whereas bedtime toothbrushing, use of fluoride toothpaste, receipt of an annual oral examination, and long-term vitamin D (≥2 years) and calcium supplementation were associated with lower odds. Third, children with ECC had less favorable growth indicators; in particular, caries experience (dmft) was inversely correlated with weight-for-height Z (WHZ), suggesting a link between caries burden and acute undernutrition.

Our prevalence and dmft estimates are broadly consistent with reports from comparable urban preschool settings, although between-study variation remains substantial. Differences in sampling frames, survey year, socio-economic context, and diagnostic thresholds likely contribute to heterogeneity across studies (24–27). In our age-stratified analyses (3–6 years), ECC prevalence and dmft did not differ significantly by age, indicating a relatively high burden throughout the preschool period rather than a monotonic age-related increase.

The observed determinants of ECC align with established mechanisms. Frequent sugar exposure promotes acidogenic biofilm activity, demineralization, and lesion progression (28). By contrast, fluoride use, regular toothbrushing, and routine dental visits reduce caries risk by enhancing remineralization, lowering plaque acidogenicity, and enabling early detection and management. Suboptimal oral-hygiene practices are common among preschool children, reinforcing the need for caregiver supervision (29). The association between long-term vitamin D supplementation and lower ECC odds is biologically plausible given vitamin D's roles in enamel development and host defense (30, 31); however, supplementation here may also serve as a proxy for health-seeking behavior, and residual confounding cannot be excluded. Parental oral-health knowledge was strongly associated with lower ECC, supporting caregiver-focused education as a practical prevention strategy (32, 33).

We explored whether ECC relates to growth and development beyond simple anthropometric description. Growth in early childhood is shaped by multiple determinants, including age, sex, infection and inflammation, diet quality and adequacy, feeding practices, sleep, socio-economic conditions, and parental education (34, 35). In our data, ECC was associated with a higher prevalence of suboptimal anthropometric outcomes, and dmft correlated negatively with WHZ. Several mechanisms may underlie this pattern: dental pain and hypersensitivity can reduce dietary intake and dietary diversity; disturbed sleep may impair growth hormone secretion; and chronic low-grade oral inflammation may affect energy balance and nutrient utilization (36). Caregiver awareness and prioritization of oral health can also influence both ECC and health-seeking behaviors relevant to growth (37). Importantly, we considered several potential confounders in our ECC analyses (age, sex, parental education, recent morbidity, feeding history including night-time feeding, sweet-food and sugar-sweetened beverage frequency, oral hygiene practices, and vitamin D/calcium supplementation). Nevertheless, our cross-sectional design precludes causal inference regarding directionality, and residual confounding (e.g., household income, food insecurity, birthweight or prematurity, physical activity) remains possible.

Nutrition is a key pathway linking oral health and growth (34). We characterized diet using a brief food-frequency module capturing cariogenic exposures (sweet foods and sugar-sweetened beverages) and proxies of diet quality (fruit/vegetables, dairy, protein-rich foods), and we recorded vitamin D and calcium supplementation through caregiver report. While these measures allowed us to account for salient dietary behaviors in relation to ECC, they do not replace comprehensive dietary assessment (e.g., 24 h recalls, energy and micronutrient intake) or biochemical indicators (e.g., serum 25-hydroxyvitamin D). Future studies should incorporate validated dietary instruments and biomarkers, and evaluate mediation by pain, sleep disturbance, and inflammation to clarify causal pathways.

This study has limitations. The cross-sectional design limits causal interpretation. Behavioral and supplementation data were caregiver-reported and subject to recall and social-desirability bias; supplementation was not verified against medical records. We lacked information on birth history, household income, and food security, and did not measure serum vitamin D. The sample was drawn from a single setting, which may limit generalizability. Our study did not capture socio-demographic factors such as parental income or education. Future research should integrate these variables to examine structural inequities in oral health prevention. Strengths include standardized WHO-based caries assessment, duplicate anthropometry with quality checks, and systematic collection of key dietary and hygiene behaviors alongside parental knowledge.

In this preschool cohort, ECC was associated with less favorable growth status, most notably a lower WHZ, and with higher prevalence of undernutrition indicators. At the same time, ECC showed strong associations with modifiable behaviors and caregiver factors, including sugar exposure, toothbrushing practices, fluoride use, routine dental visits, and long-term vitamin D and calcium supplementation. These findings support integrating caries prevention into child growth and nutrition programs, emphasizing sugar reduction, fluoride-based hygiene, regular dental check-ups, and caregiver education. Longitudinal and interventional studies with detailed dietary assessment and biomarker data are needed to clarify causality and mechanisms.

## Data Availability

The original contributions presented in the study are included in the article/Supplementary Material, further inquiries can be directed to the corresponding author.
