# Supplementary material for: Association between early childhood caries and anthropometric growth and nutritional status in preschool children: a cross-sectional study
Source: Front Pediatr. 2025 Nov 19;13:1623666. doi: 10.3389/fped.2025.1623666 (PMC12672484; doi:10.3389/fped.2025.1623666)
Supplement: Supplementary file 1 [file Datasheet1.docx]

Caregiver Questionnaire for Preschool Oral Health, Diet, and Growth

Date: [insert date]

Population: Preschool children aged 3–6 years

Respondent: Parent or primary caregiver

Mode: Self-administered or interviewer-assisted

Languages: English and Chinese

General instructions

- Recall periods: feeding history (infancy); current diet (past 3 months); oral hygiene behaviors (current routine); dental visit (past 12 months); recent morbidity (past 2 weeks); micronutrient supplementation (cumulative duration).

- Coding: Unless otherwise specified, Yes=1, No=0; Don’t know=99; Not applicable=77; Prefer not to answer=88.

- Units: Height (cm, to 0.1), Weight (kg, to 0.1), Age (months, integer).

- Skip logic: Questions with [Skip] indicate conditional routing.

Section A. Child basic information 儿童基本信息

A1. Child’s sex 性别

- 1 Male 男 2 Female 女

A2. Child’s date of birth 出生日期

- YYYY-MM-DD

A3. Child’s age at examination (months) 检查时年龄（月）

- Integer 整数

A4. Only child 是否独生子女

- 1 Yes 是 0 No 否

A5. Recent morbidity in past 2 weeks (fever, diarrhea, respiratory symptoms) 近两周是否有发热、腹泻或呼吸道症状

- 1 Yes 有 0 No 无 99 Don’t know 不清楚

Section B. Early feeding history 早期喂养史

B1. Exclusive breastfeeding during the first 6 months 前6个月是否纯母乳喂养

- 1 Yes 是 0 No 否 99 Don’t know 不清楚

B2. Total duration of breastfeeding 母乳喂养总时长

- Months 月（整数）；99 Don’t know 不清楚

B3. Bottle feeding in infancy 婴儿期是否使用奶瓶喂养

- 1 Yes 是 0 No 否 99 Don’t know 不清楚

B4. Night-time feeding during infancy (before tooth eruption) 婴儿期（牙未萌出前）是否夜间喂养

- 1 Never 从不 2 Occasionally 偶尔 3 ≥3 nights/week 每周≥3晚 99 Don’t know 不清楚

B5. Night-time feeding after tooth eruption 牙萌出后是否夜间喂养

- 1 Never 从不 2 Occasionally 偶尔 3 ≥3 nights/week 每周≥3晚 99 Don’t know 不清楚

Section C. Current diet (past 3 months) 近期饮食（过去3个月）

C1. Frequency of sweet foods (candies, cookies, cakes, chocolates) 甜食（糖果、饼干、蛋糕、巧克力）食用频率

- 0 Never 从不 1 ≤1 time/week 每周≤1次 2 2–4 times/week 每周2–4次 3 ≥5 times/week 每周≥5次 99 Don’t know 不清楚

C2. Frequency of sugar-sweetened beverages (soda, sweetened juices, sweetened milk/tea) 含糖饮料（汽水、加糖果汁、加糖奶/茶）饮用频率

- 0 Never 从不 1 ≤1 time/week 每周≤1次 2 2–4 times/week 每周2–4次 3 ≥5 times/week 每周≥5次 99 Don’t know 不清楚

C3. Fruit intake frequency 水果摄入频率（每周天数）

- 0 ≤1 day/week 每周≤1天 1 2–4 days/week 每周2–4天 2 5–7 days/week 每周5–7天 99 Don’t know 不清楚

C4. Vegetable intake frequency 蔬菜摄入频率（每周天数）

- Coding same as C3 编码同C3

C5. Dairy intake 乳制品摄入（每日份数；1份≈250 ml牛奶或等价）

- 0 <1 serving/day <1份/日 1 1 serving/day 1份/日 2 ≥2 servings/day ≥2份/日 99 Don’t know 不清楚

C6. Protein-rich foods (meat/eggs/beans) intake frequency 蛋白质类食物（肉/蛋/豆类）摄入频率（每周天数）

- 0 ≤1 day/week 每周≤1天 1 2–4 days/week 每周2–4天 2 5–7 days/week 每周5–7天 99 Don’t know 不清楚

Section D. Oral hygiene 口腔卫生行为

D1. Toothbrushing frequency 刷牙频率（每天）

- 0 <1/day 每天不足1次 1 1/day 每天1次 2 ≥2/day 每天≥2次 99 Don’t know 不清楚

D2. Bedtime toothbrushing 是否睡前刷牙

- 1 Yes 是 0 No 否 99 Don’t know 不清楚

D3. Use of fluoride toothpaste 是否使用含氟牙膏

- 1 Yes 是 0 No 否 99 Don’t know 不清楚

D4. Age at toothbrushing initiation 开始刷牙的年龄

- Years 岁（一位小数可选）；99 Don’t know 不清楚

Section E. Dental service utilization 口腔医疗服务利用

E1. Dental visit in past 12 months 过去12个月是否看牙医/口腔检查

- 1 Yes 是 0 No 否 99 Don’t know 不清楚

E2. Annual oral examination (routine check) 是否每年例行口腔检查

- 1 Yes 是 0 No 否 99 Don’t know 不清楚

Section F. Micronutrient supplementation 微量营养素补充

F1. Vitamin D supplementation 维生素D补充

- Ever used 曾经补充：1 Yes 是 0 No 否 99 Don’t know 不清楚

- Current use 当前是否在用：1 Yes 是 0 No 否 99 Don’t know 不清楚

- Cumulative duration 累计补充时长：0 <6 months <6个月；1 6–<12 months 6–<12个月；2 12–<24 months 12–<24个月；3 ≥24 months ≥24个月；99 Don’t know 不清楚

- Typical daily dose (if known) 日常剂量（若知）：IU/日；99 Don’t know 不清楚

- Source 来源：1 Drops 滴剂 2 Tablets 片剂 3 Fortified formula 强化配方奶 4 Other 其他（请注明）

F2. Calcium supplementation 钙补充

- Coding same as F1 编码同F1

Section G. Parental sociodemographic 家长社会人口学信息

G1. Relationship to child 与儿童关系

- 1 Mother 母亲 2 Father 父亲 3 Grandparent 祖辈 4 Other 其他

G2. Parental age 家长年龄

- Years 岁（整数）

G3. Parental education 家长受教育程度

- 0 Junior high or below 初中及以下 1 High school 高中/中专 2 College/University 大学/本科 3 Postgraduate 研究生及以上

G4. Self-rated oral health knowledge 家长自评口腔健康知识水平

- 0 Low 低 1 Moderate 中等 2 High 高

Section H. Parental oral-health knowledge scale (10 items) 家长口腔健康知识量表（10题）

Response options: 1 True 正确 0 False 错误 99 Don’t know 不清楚

Scoring: Correct=1; Incorrect/Don’t know=0; Sum score 0–10; Qualified ≥7.

H1. Primary teeth are important for chewing, speech, and guiding permanent tooth eruption. 乳牙对咀嚼、发音及恒牙萌出引导很重要。

- Correct answer 正确答案：True 正确

H2. Night-time feeding after tooth eruption increases the risk of caries. 牙长出后仍夜间喂养会增加龋风险。

- Correct: True

H3. Toothbrushing before bedtime helps prevent caries. 睡前刷牙有助于预防龋齿。

- Correct: True

H4. Fluoride toothpaste helps prevent caries in children. 含氟牙膏有助于预防儿童龋齿。

- Correct: True

H5. Sweet foods and sugary drinks are major risk factors for caries. 甜食和含糖饮料是造成龋齿的主要危险因素。

- Correct: True

H6. A dental check-up once a year is recommended for preschool children. 学龄前儿童建议每年至少检查一次口腔。

- Correct: True

H7. If a primary tooth has caries, treatment is unnecessary because it will be replaced. 乳牙龋坏无需治疗，因为会被替换。

- Correct: False 错误

H8. White spot lesions on enamel can progress to cavities if not managed. 牙釉质白斑若不处理可能发展成龋洞。

- Correct: True

H9. Vitamin D has a role in tooth and bone mineralization. 维生素D参与牙齿和骨骼的矿化。

- Correct: True

H10. Caries is caused only by bacteria, not by sugar intake. 龋齿仅由细菌引起，与糖摄入无关。

- Correct: False 错误

Section I. Definitions and derived variables 定义与派生变量

I1. ECC case definition ECC病例定义

- ECC=1 if dmft ≥ 1; ECC=0 if dmft=0.

I2. dmft components dmft组成

- d = cavitated dentine-level caries, including restorations with secondary caries; m = missing due to caries; f = filled without secondary caries.

I3. Sugar exposure variables 糖暴露变量

- Sweet foods frequency (C1): ordinal 0–3; SSB frequency (C2): ordinal 0–3.

- Optional composite: High sugar exposure=1 if C1≥2 or C2≥2; else 0.

I4. Diet quality proxies 饮食质量代理指标

- Fruit/vegetables: “adequate”=1 if C3≥2 and C4≥2; else 0.

- Dairy: “adequate”=1 if C5≥2; else 0.

- Protein-rich foods: “adequate”=1 if C6≥2; else 0.

I5. Oral hygiene behaviors 口腔卫生行为

- Bedtime brushing (D2): binary; Fluoride toothpaste (D3): binary.

I6. Dental utilization 口腔服务利用

- Annual oral exam (E2): binary.

I7. Supplementation variables 营养素补充变量

- Vitamin D duration categories: F1 duration coded 0–3; binary long-term VD=1 if ≥24 months; else 0.

- Calcium duration categories: F2 duration coded 0–3.

I8. Anthropometric variables 人体测量变量

- Age in months (A3); height cm; weight kg.

- HAZ, WAZ, WHZ computed via WHO standards; stunting/underweight/wasting defined as Z<-2.

I9. Confounders 混杂因素

- Include age, sex, parental education, recent morbidity, diet frequencies, oral hygiene, supplementation.

Section J. Enumerator notes and quality control 调查员说明与质控

J1. Environment 环境

- Ensure adequate lighting; quiet setting; caregiver privacy.

J2. Clarifications 术语说明

- Sugar-sweetened beverages include drinks with added sugar: sodas, sweetened juices, sweetened milk/tea. 不含纯水、不加糖的纯牛奶或无糖茶。

- Night-time feeding refers to feeding after the child has fallen asleep or during night awakenings. 夜间喂养指孩子入睡后或夜间醒来时的喂养。

J3. Consistency checks 一致性检查

- Cross-check D3 (fluoride toothpaste) if child uses “toddler toothpaste” without label; mark 99 if uncertain.

- For supplementation dose unknown, code as 99.

J4. Missing data 缺失处理

- If any core variable missing (A3, height, weight), mark as incomplete and do not include in growth analyses.

J5. Data entry 数据录入

- Double entry with validation; range checks: age 36–83 months; height 85–130 cm; weight 10–35 kg; flag outliers for verification.

Consent and confidentiality 知情与保密

- This questionnaire is for research purposes. Participation is voluntary. Responses are confidential and analyzed in aggregate. 本问卷仅用于科研，参与自愿，信息保密，仅以汇总形式分析。
